# Deubiquitinase Ubp5 Is Required for the Growth and Pathogenicity of *Cryptococcus gattii*

**DOI:** 10.1371/journal.pone.0153219

**Published:** 2016-04-06

**Authors:** Yunfang Meng, Chao Zhang, Jiu Yi, Zhaojing Zhou, Zhenzong Fa, Jingyu Zhao, Yali Yang, Wei Fang, Yan Wang, Wan-qing Liao

**Affiliations:** 1 Shanghai Key Laboratory of Molecular Medical Mycology, Shanghai Institute of Medical Mycology, Second Military Medical University, Shanghai, China; 2 PLA Key Laboratory of Mycosis, Department of Dermatology and Venereology, Changzheng Hospital, Shanghai, China; 3 Shanghai Dermatology Hospital, Shanghai, China; 4 Department of Pharmacology, School of Pharmacy, Second Military Medical University, Shanghai, China; 5 Department of Dermatology, Shandong Provincial Hospital Affiliated to Shandong University, Jinan, Shandong, China; University of Minnesota, UNITED STATES

## Abstract

*Cryptococcus gattii* is a resurgent fungal pathogen that primarily infects immunocompetent hosts. Thus, it poses an increasingly significant impact on global public health; however, the mechanisms underlying its pathogenesis remain largely unknown. We conducted a detailed characterization of the deubiquitinase Ubp5 in the biology and virulence of *C*. *gattii* using the hypervirulent strain R265, and defined its properties as either distinctive or shared with *C*. *neoformans*. Deletion of the *C*. *gattii* Ubp5 protein by site-directed disruption resulted in a severe growth defect under both normal and stressful conditions (such as high temperature, high salt, cell wall damaging agents, and antifungal agents), similar to the effects observed in *C*. *neoformans*. However, unlike *C*. *neoformans*, the *C*. *gattii ubp5*Δ mutant displayed a slight enhancement of capsule and melanin production, indicating the evolutionary convergence and divergence of Ubp5 between these two sibling species. Attenuated virulence of the *Cg-ubp5*Δ mutant was not solely due to its reduced thermotolerance at 37°C, as shown in both worm and mouse survival assays. In addition, the assessment of fungal burden in mammalian organs further indicated that Ubp5 was required for *C*. *gattii* pulmonary survival and, consequently, extrapulmonary dissemination. Taken together, our work highlights the importance of deubiquitinase Ubp5 in the virulence composite of both pathogenic cryptococcal species, and it facilitates a better understanding of *C*. *gattii* virulence mechanisms.

## Introduction

Cryptococcosis is one of most prominent invasive fungal diseases; it can invade both immunocompromised and immunocompetent hosts and often manifests as life-threatening meningoencephalitis. Among its two major pathogenic agents, *Cryptococcus neoformans* (Cn) is known to mainly infect the immunocompromised population and is responsible for the vast majority of cases of cryptococcosis globally[1]. The other agent, *Cryptococcus gattii* (Cg), was originally believed to be restricted to healthy individuals in tropical and subtropical countries such as Australia and Papua New Guinea[2, 3]. The outbreak of *C*. *gattii* cryptococcosis in temperate regions such as Vancouver Island, British Columbia, and the Pacific Northwest has redrawn public attention to this resurgent fungal pathogen[4, 5].

As the sibling species of *C*. *neoformans*, *C*. *gattii* is also an encapsulated budding yeast, but it exhibits distinct morphological, biochemical, and ecological patterns. For example, *C*. *gattii* yields both round and bacilliform cells, and it is consistently found inhabiting decaying trees but not bird droppings like *C*. *neoformans*[6, 7]. Although these pathogens are not routinely discriminated in clinical practice, their interspecific differences are significant for the clinical manifestation and management of infection. Brain infection caused by *C*. *gattii* is associated with an increased number of cryptococcomas, more neurological complications, and a slower response to therapy, and it usually requires additional diagnostic follow-ups and more frequent neurosurgical intervention, as compared with infection with *C*. *neoformans*[8]. The unique pattern of *C*. *gattii* in terms of its epidemiological and clinical features may be largely due to its distinctive mechanisms of pathogenesis. Previous studies have suggested that *C*. *gattii* infection results in defective induction of host immune responses, such as the arrested migration of neutrophils and the reduced expression of several protective pro-inflammatory cytokines[9, 10]. Furthermore, *C*. *gattii* also displays some divergent virulence-regulatory mechanisms compared with *C*. *neoformans*, such as the antioxidant superoxide dismutase (Sod1) and trehalose-6-phosphate synthase (Tps1 and Tps2)[11–14]. It is clear that *C*. *gattii* may rely on the variegated expression of virulence genes or some unknown but unique virulence traits to adapt to the host environment *in vivo*. A complete understanding of its unique mechanisms of pathogenesis is essential for allowing an accurate diagnosis and more appropriate intervention strategies in *C*. *gattii* infection, and these mechanisms remain to be further elucidated.

Ubiquitination is a critical reversible post-translational modification for regulating cell growth and physiology in eukaryotes[15]. Ubiquitin homeostasis is mainly determined by the processing of its precursors and its recycling from substrates via deubiquitinases (DUBs). DUBs are a conserved superfamily of proteases that are involved in a variety of biological processes, such as the cell cycle, signal transduction, and the stress response[16], and they have recently emerged as attractive targets in anticancer therapy[17]. For example, the deubiquitinase Usp7 has been linked to human hematopoietic tumors based on its ability to regulate the degradation of the tumor suppressor p53[18]. In model fungi, DUBs have also been reported to be essential for several cellular functions such as nutrient sensing, sexual reproduction, and stress responses[19–21]. However, few studies have reported the roles of deubiquitinase in the virulence of human fungal pathogens. Using a systematic genetic analysis, Liu *et al*. first demonstrated that some DUBs might be involved in melanization and pathogenesis in *C*. *neoformans*[22]. Thus, from the remaining DUBs, we further identified Ubp5, which is essential for sexual reproduction, the stress response, and the virulence composite in *C*. *neoformans*[23]. Interestingly, the same deubiquitinase gene has also been shown to be up-regulated in several hypervirulent *C*. *gattii* isolates from the Vancouver Island outbreak, the expression profiles of which display a significant correlation with the cryptococcal intracellular proliferation rate inside macrophage-like cells[24]. Hence, we hypothesize that deubiquitinase Ubp5 may possess a divergent function in the pathogenesis of *C*. *gattii*.

In the present study, we evaluated the biological functions of Ubp5 in *Cryptococcus gattii* using the hypervirulent strain R265 as a model. Deletion of Ubp5 in *C*. *gattii* revealed a severe growth defect under both normal and stressful conditions, and it also attenuated virulence in non-vertebrate and mammalian hosts. In contrast to the findings for *C*. *neoformans*, enhanced capsule production and melanin synthesis were observed in the *C*. *gattii ubp5*Δ mutant, indicating that the utilization of Ubp5 has evolved for distinct regulatory purposes in the virulence composite of these sibling species. Taken together, our study demonstrates the functional convergence and divergence of Ubp5 among pathogenic *Cryptococcus* species, facilitating a better understanding of *C*. *gattii* virulence mechanisms.

## Results

### Characterization of the *C*. *gattii* gene *UBP5*

The *C*. *gattii* gene *UBP5* (CNBG_6153) displayed approximately 87% nucleotide identity to *UBP5* from *C*. *neoformans* var. *grubii* (CNAG_05650) or *C*. *neoformans* var. *neoformans* (CNBL2960). A phylogenetic analysis of the protein alignment was performed using the deubiquitinase Ubp5 orthologs of the *C*. *neoformans* species complex and 10 other fungal species. This protein was classified into distinct clades of basidiomycetous yeasts, ascomycetous yeasts, molds, and zygomycetous molds, consistent with their evolutionary relationship (S1 Fig). Among the basidiomycetes, *C*. *neoformans* var. *grubii* and *C*. *neoformans* var. *neoformans* belonged to the same species, which was distinct from *C*. *gattii*. Interestingly, the Ubp5 orthologs of *C*. *gattii* and *C*. *neoformans* var. *grubii* formed one sister clade, suggesting an evolutionary divergence among the pathogenic cryptococcal species. Analysis of the predicted *C*. *gattii* protein Ubp5 revealed the presence of MATH (amino acids 55 to 206), UCH (amino acids 208 to 525), and USP7 (amino acids 631 to 1103) motifs. These motifs and their arrangement were common in Ubp5 orthologs from all of the analyzed fungi, and the three domains displayed identities of approximately 98%-99% in the *C*. *neoformans* species complex, indicating that the protein structure of deubiquitinase Ubp5 was evolutionarily conserved.

### Ubp5 is required for cell propagation of *C*. *gattii*

**Fig 1:**
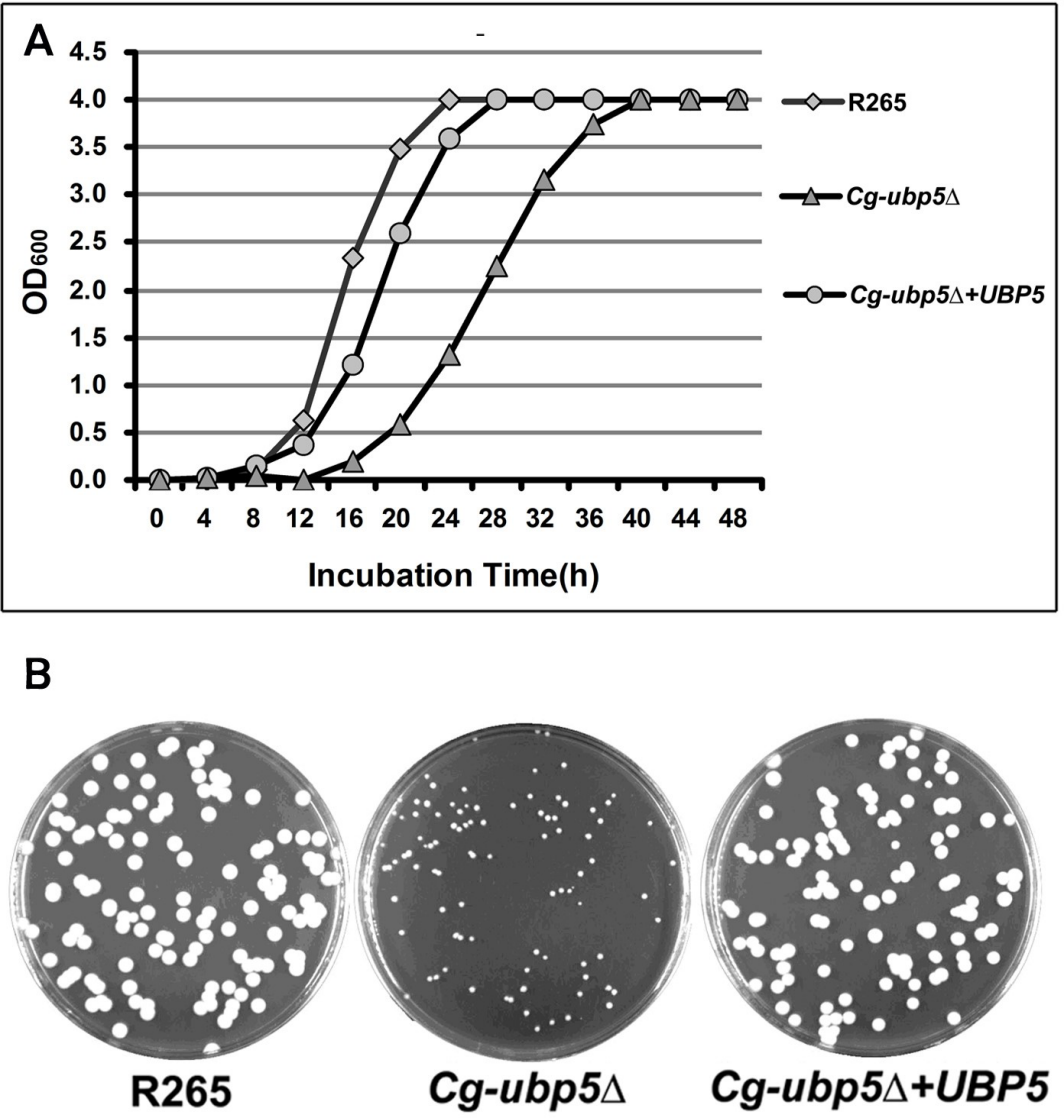
Growth curve assay and colony size assessment. A. The WT, mutant, and reconstituted strains were grown in YPD broth overnight at 30°C. Next, 10^6^ cells of each strain were transferred to 30 mL fresh YPD broth in flasks and incubated at 30°C. The OD_600_ values were measured for each group at four-hour intervals. The growth rate of the *Cg-ubp5*Δ mutant was significantly reduced compared with the other two strains. B. One hundred cells from each strain were spread onto YPD agar, incubated for 5 days at 30°C and photographed.

To determine the biological functions of deubiquitinase Ubp5 in *C*. *gattii*, we constructed the *Cg-ubp5*Δ mutant and its reconstituted strain *Cg-ubp5*Δ*+UBP5* on the background of the R265 hypervirulent isolate. Similarly to *C*. *neoformans*[23], the lack of Ubp5 significantly delayed the growth of *C*. *gattii*, even on rich medium at 30°C. As shown in Fig 1A, the *Cg-ubp5*Δ mutant required an incubation of approximately 40 hours to achieve stationary phase, while the WT strain rapidly entered stationary phase by 24 hours. The reconstituted strain of *CgUBP5* displayed a partially restored growth rate similar to the WT strain. We also compared the colony sizes of these three strains after a five-day incubation on YPD agar at 30°C. The remarkable differences in colony size further confirmed the decreased growth rate exhibited by the *Cg-ubp5*Δ mutant (Fig 1B), suggesting that Ubp5 was involved in the propagation of *C*. *gattii*.

### *C*. *gattii* Ubp5 is involved in fungal tolerance to multiple stressors *in vitro*

**Fig 2:**
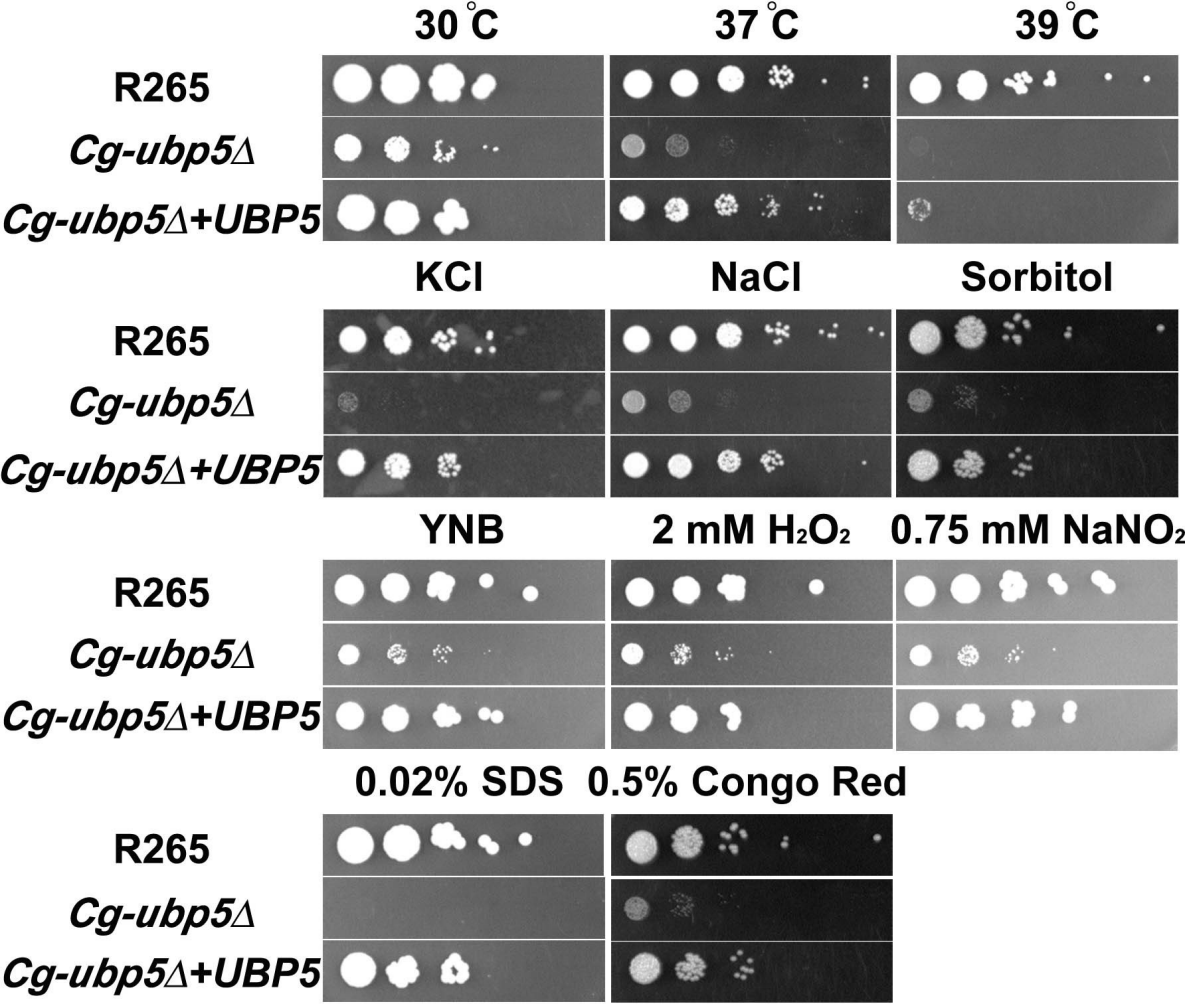
*C*. *gattii* Ubp5 is involved in fungal responses to various stressors. The R265, *Cg-ubp5*Δ, and *Cg-ubp5*Δ*+UBP5* strains were grown on YPD broth to saturation at 30°C and then serially diluted 10-fold (1–10^6^ dilutions). 3 μL suspension of 10^8^ cells/mL were spotted on YPD or YNB agar (containing different stress-inducing agents), incubated for five days and photographed.

Next, we evaluated the effect of *UBP5* disruption on stress responses in *C*. *gattii*. Similarly to the *Cn-ubp5*Δ mutant[23], the *Cg-ubp5*Δ mutant strains displayed enhanced susceptibility to various stressors *in vitro* (Fig 2). The results revealed that the *Cg-ubp5*Δ mutant was hypersensitive to high temperature, exhibiting a partial growth defect at 37°C and complete fungistasis at 39°C. Similar phenotypes were also observed in the mutant strain following exposure to osmotic shock or cell membrane/wall damaging agents. In response to oxidative and nitrosative damage, the *Cg-ubp5*Δ mutant strains exhibited slight sensitivity, but it did not differ from the effects observed on YNB medium, suggesting that Ubp5 might not be directly involved in stress tolerance to peroxide and nitric oxide in *C*. *gattii*. Contrasting results have been obtained in *C*. *neoformans*, in which Ubp5 was essential for cryptococcal resistance to both H_2_O_2_ and NO[23]. In addition, we also tested the impact of the deletion of *UBP5* on the susceptibility of *C*. *gattii* to several antifungal drugs. In comparison to the WT strain, the *Cg-ubp5*Δ mutant strains exhibited a 2-fold reduction in the MIC for amphotericin B and at least a 4-fold reduction in the MICs of other common antifungal agents such as flucytosine, terbinafine, and azoles (Table 1). Interestingly, reconstitution of *Cg-UBP5* failed to restore the survival of *C*. *gattii* at 39°C in spite of restoring its tolerance to the other *in vitro* stressors, which might be due to damage caused by ectopic integration and/or repeated biolistic transformations. These data indicate that deubiquitinase Ubp5 positively regulates the fungal stress response, but with a subtle distinction, in both *C*. *neoformans* and *C*. *gattii* species.

**Table 1:** Antifungal susceptibility assay.

Strains	MICs (μg/mL)					
	Amphotericin B	Fluconazole	Flucytosine	Itroconazole	Terbinafine	Voriconazole
**R265**	0.5	16	32	0.5	2	0.25
***Cg-ubp5***Δ	0.25	4	8	0.0625	0.25	0.0313
***Cg-ubp5***Δ***+UBP5***	0.5	16	32	0.5	4	0.25
**ATCC22019**	1	0.5	0.5	0.25	8	0.06

### Deletion of *UBP5* enhances capsule and melanin production in *C*. *gattii*

**Fig 3:**
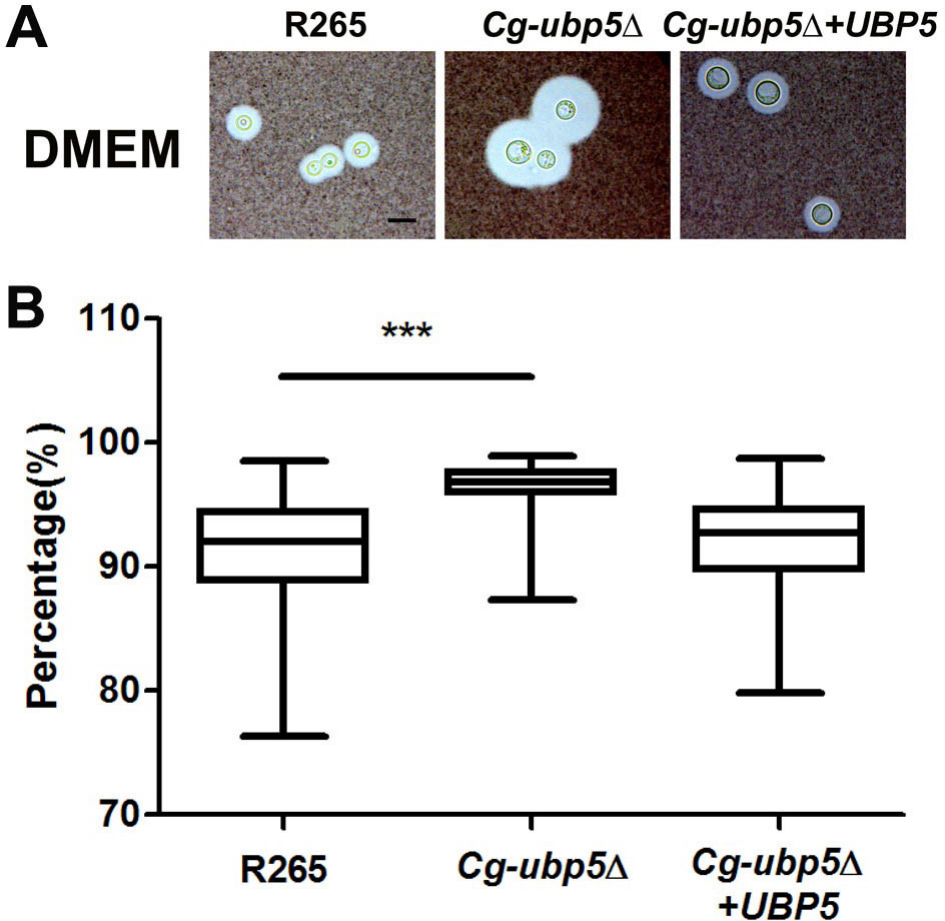
*UBP5* mutation enhances capsule production in *C*. *gattii*. A. The WT, mutant, and reconstituted strains were incubated on DMEM medium for capsule induction at 37°C for three days. Capsules were assessed by India ink staining and visualization at 100X magnification (scale bar = 10 μm). B. Relative capsule volume on DMEM medium. Relative capsule volume = (Total Volume–Packed Volume)/Total Volume (N = 50). Wilcoxon test was performed to examine the capsule difference between R265 and *Cg-ubp5*Δ strains. The results revealed enhanced capsule production in the *Cg-ubp5*Δ mutant strain (*P*<0.01).

**Fig 4:**
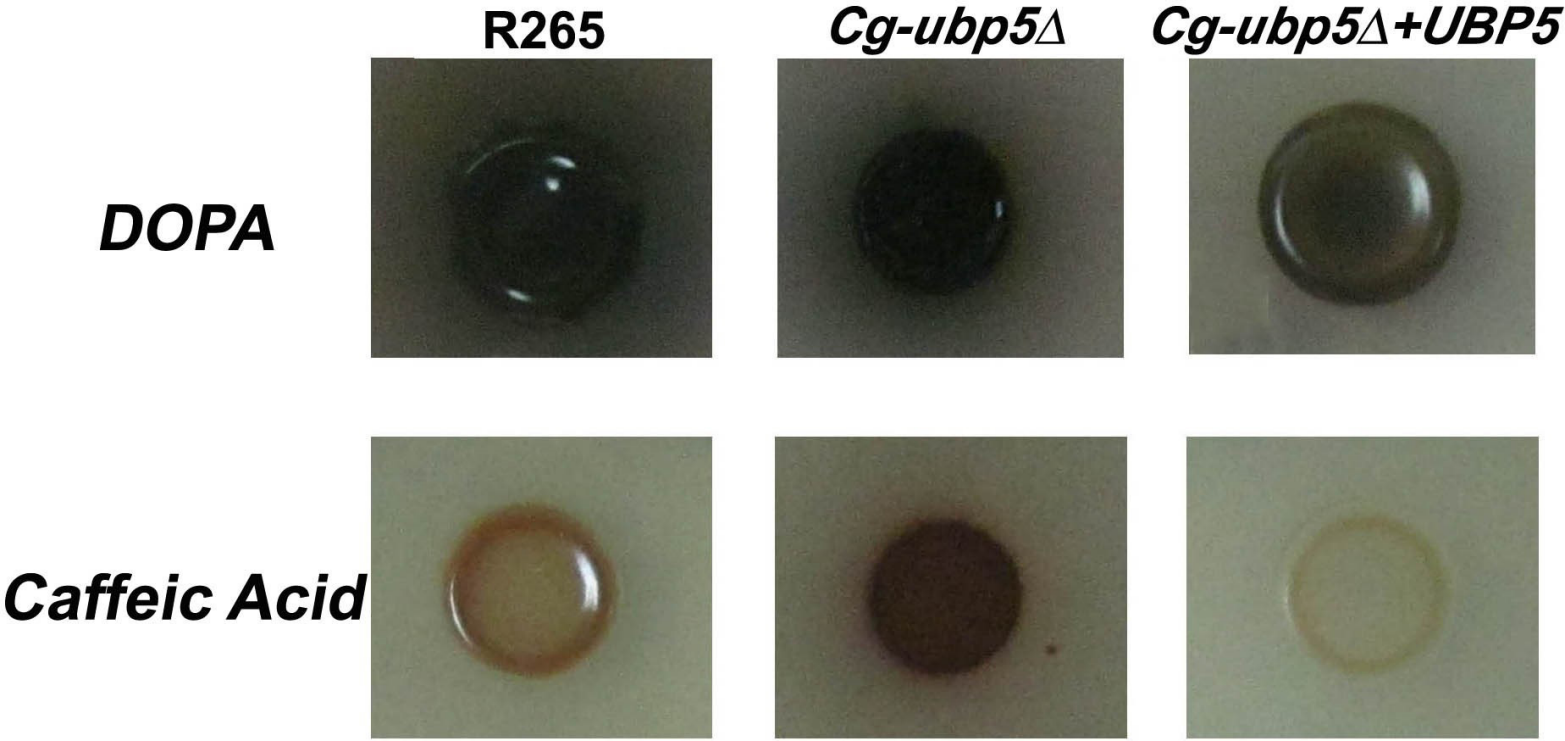
Ubp5 negatively regulates melanin production in *C*. *gattii*. Strains grown in YPD broth were washed twice with PBS buffer, and a 5 μL suspension of 10^7^ cells/mL was spotted on L-DOPA and Caffeic Acid media and incubated for 5 days at 30°C for melanin induction.

We tested whether the deletion of *UBP5* could influence other known pathogenic factors, such as the polysaccharide capsule and melanin production in *C*. *gattii*. Unlike the *Cn-ubp5*Δ mutant[23], deletion of Ubp5 led to a slight enlargement in the size of the *C*. *gattii* capsule in DMEM when grown in the presence of CO_2_ (Fig 3A). A minimum of 50 cells from each strain were measured, and the average capsule size (relative volume) of the *Cg-ubp5*Δ strain was 96.2% compared with 91.0% and 92.3% in the WT and reconstituted strains, respectively (Fig 3B, *P*<0.001). Furthermore, deletion of Ubp5 had a similar effect on the pathogenic factor laccase (Fig 4). The *C*. *gattii ubp5*Δ strain displayed slight hypermelanization and produced leaky melanin around the colonies compared with the WT strain when incubated on L-DOPA medium at 30°C for 5 days. A similar difference in melanin production was more evident on caffeic acid medium, which revealed a sharp distinction in the melanin phenotype due to Ubp5 deletion in *C*. *neoformans*. We also noted that the complemented strain showed less melanization than the WT strain in both L-DOPA and caffeic acid, which might be attributed to trancriptional alteration of *UBP5* and/or secondary mutation caused by ectopic integration. Taken together, these data establish a distinct role for Ubp5 in capsule or melanin production in *C*. *gattii*.

### Role of Upb5 on *C*. *gattii* parasitism inside macrophages

**Fig 5:**
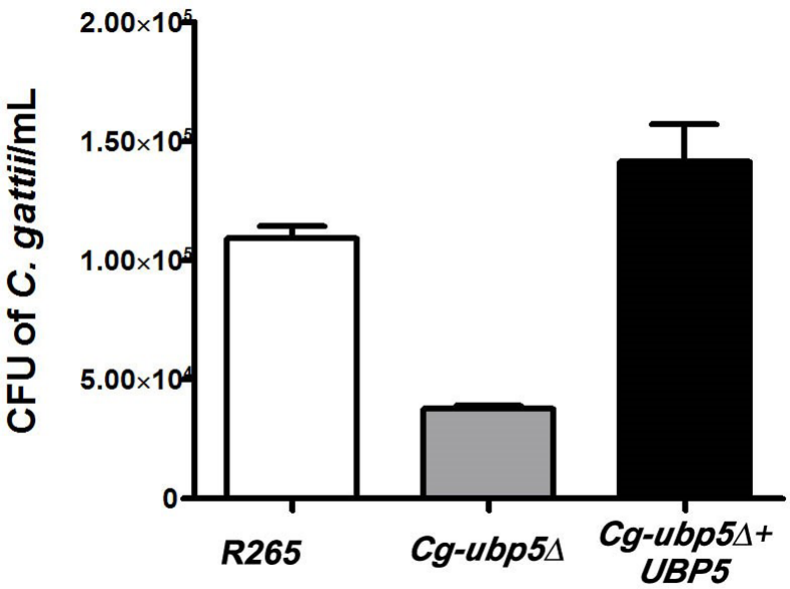
Ubp5 mediates the survival and proliferation of *C*. *gattii* in macrophages. Activated J774A.1 macrophages were infected with R265, *Cg-ubp5*Δ, and *Cg-ubp5*Δ*+UBP5* strains of *C*. *gattii*. After 2 hours of coincubation at 37°C with 5% CO_2_, the extracellular yeasts were removed, and the co-cultures were incubated for 24 hours under the same conditions. The macrophages were lysed, and the samples were then incubated on YPD agar at 30°C for 4 days to quantify the cryptococcal colonies. Each strain was assayed four times (average ± SEM, *P*<0.0001).

*Cryptococcus* spp. have been generally accepted as facultative intracellular pathogens, and the hypervirulence of some *C*. *gattii* strains has been closely associated with their potent proliferative capacity inside macrophages[25, 26]. Thus, we assessed the ability of the *Cg-ubp5*Δ mutant to parasitize macrophages by co-incubating them with activated macrophages. Co-culture with activated J774A.1 macrophages revealed a 65.7% reduction in the intracellular survival of the mutant strain after 24 h compared with the background strain R265 (*P*<0.0001) (Fig 5). Reconstitution of *CgUBP5* completely restored the intracellular proliferation of the mutant inside macrophages. Similar results were obtained in repeated experiments, suggesting that deubiquitinase Ubp5 is essential for *C*. *gattii* survival inside macrophages.

### *C*. *gattii* Upb5 is essential for virulence in mammals

**Fig 6:**
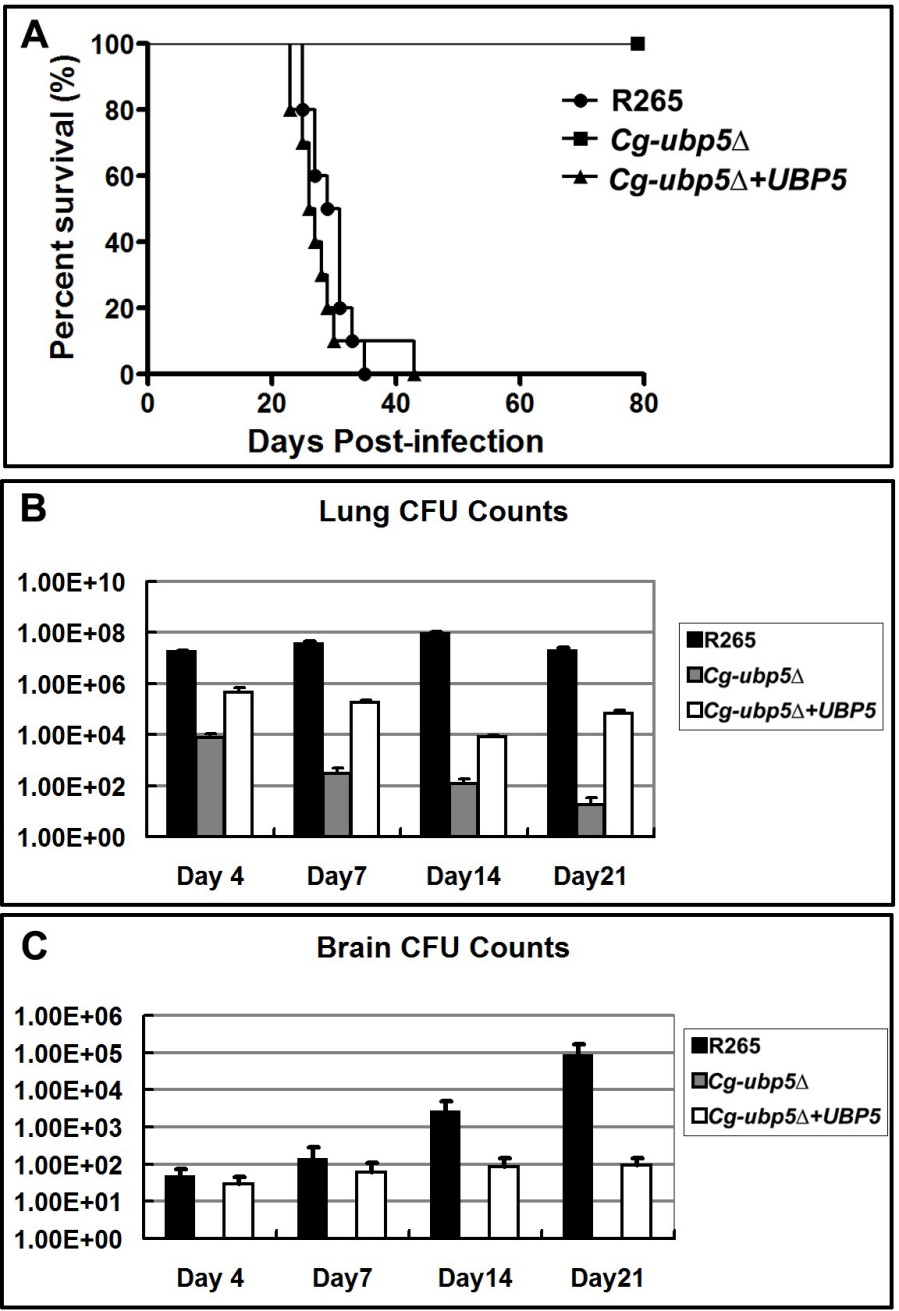
Deletion of Ubp5 attenuates the virulence of *C*. *gattii* in a murine inhalation model. A. Survival curve of mouse inhalational cryptococcosis with R265, *Cg-ubp5*Δ, and *Cg-ubp5*Δ*+UBP5* strains over 80 days. All of the mice infected with R265 and *Cg-ubp5*Δ*+UBP5* were sacrificed, but all of the mice in the *Cg-ubp5*Δ group survived (*P*<0.001). B&C. Fungal burden in the lung and brain. Organs were removed at 4, 7, 14, and 21 days post-infection in the three groups.

To gain insight into the overall impact of the *C*. *gattii* Ubp5 deletion on the total virulence composite, we first performed a survival assay using the murine inhalation model. Immunocompetent BALB/c mice were inoculated intranasally with 10^5^ cells of R265, the *Cg-ubp5*Δ strain, or the reconstituted strain *Cg-ubp5*Δ*+UBP5*. As shown in Fig 6A, mice infected with the WT strain R265 survived for 33 days, and the average survival time was 29±3.73 days. The group infected with the reconstituted strain displayed a similar survival pattern, in which all of the mice were sacrificed by day 43, and the average survival time was 28±5.75 days (*P* = 0.157). In contrast, mice in the *Cg-ubp5*Δ mutant group did not die even at 80 days after infection, suggesting a significant attenuation of *C*. *gattii* virulence due to the deletion of Ubp5 (*P*<0.001).

To investigate the potential impact of Ubp5 deletion on alveolar delivery or cryptococcal migration from the lungs, we next evaluated fungal burdens in the lung and brain in the above three groups at 4, 7, 14, and 21 days post-infection. Total lung CFU analyses at different time points post-infection revealed a high cryptococcal burden in the WT group (Fig 6B & 6C). However, the *C*. *gattii* strain lacking *UBP5* resulted in significantly reduced pulmonary fungal burden at different time points after infection (*P*<0.001), which also displayed a gradual downward trend with an extended duration of infection. Furthermore, no viable colonies were found in the brain of mice infected with the *Cg-ubp5*Δ strain, in contrast to the other groups. The mice infected with reconstituted strain *Cg-ubp5*Δ*+UBP5* displayed a slight reduction in CFU in both the lung and brain compared with the WT strain but a significant increase in CFU compared with the *Cg-ubp5*Δ strain (*P*<0.001, Fig 6B & 6C), suggesting that ectopic integration of *UBP5* partially restored the virulence of the *ubp5Δ* mutant. These data indicate that *C*. *gattii* requires Ubp5 to parasitize the lung and disseminate into other organs, especially the central nervous system.

### Impact of Upb5 on the *C*. *elegans* model

**Fig 7:**
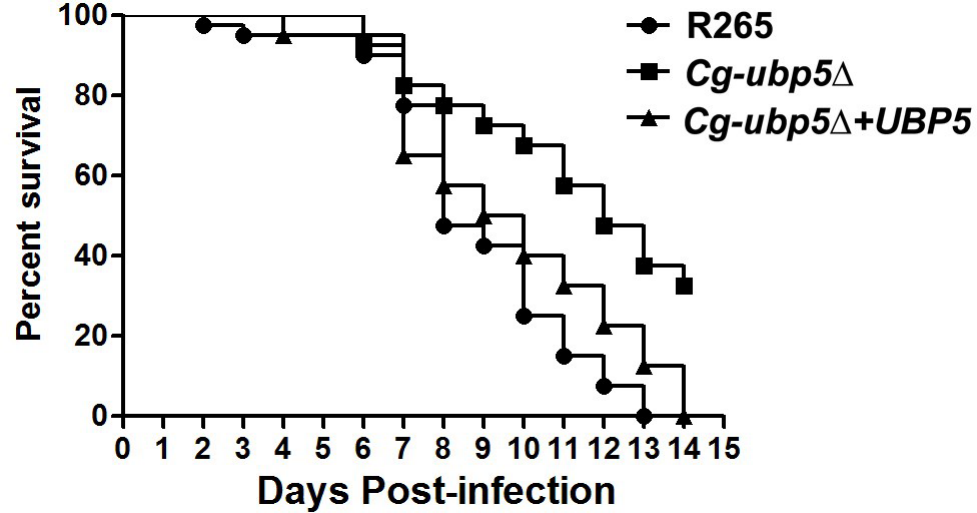
Survival analysis in the *C*. *elegans* model. Forty *C*. *elegans* per group were fed on lawns of the WT, mutant, or reconstituted strain. Ubp5 deletion attenuated the virulence of *C*. *gattii* (*P*<0.001).

Since deletion of *UBP5* enhanced the susceptibility of *C*. *gattii* to high temperature, we wondered whether attenuated *in vivo* virulence of *ubp5*Δ mutant was only attributed to its reduced thermotolerance. *Caenorhabditis elegans* provides an important model of pathogenesis at room temperature that can be utilized to exclude the potential effect of high temperature sensitivity on cryptococcal virulence[27, 28]. In the *C*. *elegans/C*. *gattii* system, the *Cg-ubp5*Δ mutant (LT_50_ = 12 days) was less virulent than the WT (LT_50_ = 8 days) or reconstituted (LT_50_ = 9 days) strains, as determined by survival analysis (*P*<0.001, Fig 7). This finding was consistent with results obtained in the murine inhalation model with these strains. Our data suggest that the lack of *UBP5* may attenuate the virulence of *C*. *gattii* independently of its influence on high-temperature tolerance.

## Discussion

*C*. *gattii* is known as the major cryptococcal pathogen in immunocompetent hosts worldwide other than in China[1, 28]. This pathogen, which was previously considered to be endemic in tropical and sub-tropical regions, has received global scientific interest due to its association with fatal outbreaks in humans and mammals, as well as its expanding geographical range[5, 29]. Experimental studies investigating the mechanisms underlying the pathogenicity of *C*. *gattii* are scarce. In the present study, we conducted a detailed characterization of the deubiquitinase Ubp5 in the biology and virulence of *C*. *gattii* using the hypervirulent strain R265, and we defined its properties as either distinctive or shared with *C*. *neoformans*.

The first phenotype observed for the *Cg-ubp5*Δ strain was its poor growth performance under both stressful and normal conditions. Similarly to its function in *C*. *neoformans*, the deubiquitinase Ubp5 of *C*. *gattii* positively regulated its responses to a variety of stressors *in vitro*, such as high temperature, high salt content, and antifungal drugs, among others. There may be several explanations for the role of Ubp5 in stress responses. First, many misfolded or damaged proteins accumulate inside the cryptococcal cell following prolonged exposure to various stressors, and this phenomenon relies in part on the ubiquitin-mediated degradation pathway for the maintenance of cellular homeostasis. The ubiquitin-proteasome pathway is critical for regulating various cellular processes, especially the stress response, in various eukaryotic species such as *Saccharomyces*. *cerevisiae*, *Schizosaccharomyces*. *pombe*, and *Candida*. *candida*[30–32]. In *C*. *neoformans*, several ubiquitin-system genes, such as *UBI4* (polyubiquitin) and *UBC6* (ubiquitin conjugating enzyme), also display significant transcriptional changes under stressful conditions[33, 34]. As a core component of the ubiquitin-proteasome system, deubiquitinase is essential for maintaining the dynamic balance of ubiquitin by processing ubiquitin precursors or proofreading ubiquitin-protein conjugation, and thus, it participates in the stress responses of fungi[35, 36]. Second, ubiquitination and deubiquitination might be an important modification mechanism in some signaling pathways associated with the stress response in fungi. For example, the HOG pathway is negatively regulated via ubiquitin-mediated degradation of the upstream component Ssk1 in *S*. *cerevisiae*[37]. In *C*. *neoformans*, many genes encoding ubiquitin-conjugating enzymes are significantly up-regulated in some HOG pathway mutants, while some genes encoding components (such as MAPKKK and Cpb1) of the MAPK and Ca^2+^/calcineurin signaling pathways also display significant transcriptional changes in the *Cn-ubp5*Δ mutant[23, 33]. The relationship of deubiquitinase with various signaling pathways remains to be further illuminated in *Cryptococcus* spp. Finally, the reduced stress tolerance of the *Cg-ubp5*Δ mutant was also associated with its slower proliferation rate, even in rich media. Yeast cell growth is a complex biological process that relies on the coordination of multiple factors, such as cell division, cell size, nutrients and energy metabolism[38, 39]. In *S*. *cerevisiae*, deubiquitination is an important modification mechanism that is involved in energy uptake and the cell cycle[21, 40]. Cg-Ubp5 might exploit similar strategies to regulate cell growth in *C*. *gattii*.

However, deletion of the deubiquitinase Ubp5 led to subtle differences in fungal susceptibility to some stressors between *C*. *gattii* and *C*. *neoformans*. For example, the *C*. *gattii ubp5*Δ strain was less sensitive to oxidative and NO stress but was more susceptible to cell wall/membrane damaging agents compared with the *Cn-ubp5*Δ mutant in *C*. *neoformans*[23]. *Cryptococcus* spp. rely on multiple signaling pathways and regulatory mechanisms to respond to each stressor[41], and the deubiquitinase Ubp5 might act on distinct substrates in its two major pathogens to differentially regulate the stress response. However, it might also be due to the functional similarities and redundancies of the DUB protein family such that selective environmental pressure could drive the microevolution of the function of Ubp5 in *Cryptococcus* spp.

The functional difference in Ubp5 between the two sibling species was more significant in terms of the expression of other pathogenic factors. In *C*. *neoformans*, CnUbp5 positively regulated both melanin and capsule production, which might be associated with its roles in regulating copper ion metabolism or polysaccharide attachment to the cell wall[23]. Interestingly, deletion of CgUbp5 led to opposite phenotypes (enhanced melanin and capsule production) in *C*. *gattii*, which further confirmed the functional reconfiguring of the homologous gene in cryptococcal evolution. *Cryptococcus* spp. exploit a similar mechanism for both capsule and melanin production, in which vesicles containing the protein components are excreted into the extracellular space and the components are then attached to the cell wall[42–45]. Several factors that participate in cell wall remodeling, such as chitin and chitosan, are also involved in capsule or melanin assembly[46–48]. For example, lack of chitosan in *C*. *neoformans* led to a "leaky melanin" phenotype like *Cg-ubp5*Δ mutant in this study[48]. Considering the hypersusceptibility of the *Cg-ubp5*Δ mutant to Congo Red, we speculate that CgUbp5 might indirectly regulate capsule or melanin production via its role in cell wall synthesis; however, this hypothesis requires further exploration.

The *Cryptococcus neoformans* species complex utilizes multiple pathogenic factors to overcome the hostile environment *in vivo* and cause damage to the host[49]. Each pathogenic factor potentially provides a different relative contribution to the overall virulence phenotype of this organism[50]. In *C*. *gattii*, deletion of deubiquitinase CgUbp5 resulted in significantly attenuated virulence in a mammalian host, although the *Cg-ubp5*Δ mutant displayed slightly enhanced production of capsule and melanin. Excluding the effect of the reduced thermotolerance, the survival assay in *C*. *elegans* model further confirmed the role of CgUbp5 in regulating the virulence composite of *C*. *gattii*. The results of our study suggest that the deubiquitinase Ubp5 is essential for the overall virulence phenotype in both *C*. *gattii* and *C*. *neoformans* but with some common and/or specialized mechanisms[23].

It is believed that cryptococcal spores or desiccated yeast cells are first inhaled into the lungs and then disseminate into extrapulmonary regions in the central nervous system when the host is immunocompromised[51, 52]. In the present study, the fungal burden in a murine model suggested that *C*. *gattii* lacking deubiquitinase Ubp5 was easily cleared by host pulmonary defense responses. This phenomenon was closely associated with the decreased survival rate of the *Cg-ubp5*Δ mutant inside macrophages. Alveolar macrophages have been demonstrated to be the first line of host defense, a primary haven for latent infection, and also to function as a Trojan horse for hematogenous dissemination during cryptococcal infection[25]. Enhanced intracellular replication within host macrophages is an important feature of the hypervirulent *C*. *gattii* strains recovered from the Vancouver Island outbreak, which are characterized by the upregulation of multiple genes including *CgUBP5*[24]. Consistent with this perspective, our work further indicates that CgUbp5 is required for the survival of *C*. *gattii* in the pulmonary space and, thus, its extrapulmonary dissemination.

In summary, our work supports the importance of deubiquitinase Ubp5 in the virulence composite of *C*. *gattii* strain R265. Ubp5 was found to be involved in cellular propagation, the stress response, capsule and melanin production, and thus pathogenicity of *C*. *gattii* in both non-vertebrate and vertebrate models. Furthermore, our results revealed the evolutionary convergence and divergence of Ubp5 in these two major cryptococcal pathogens to a certain extent, suggesting that *C*. *gattii* exploit some specialized mechanisms to adapt to environments *in vivo* and *in vitro*. However, the detailed mechanism by which Ubp5 deletion affects host immunological responses during cryptococcal infection remains unknown. It will be important to explore the potential of deubiquitinase as an anticryptococcal target.

## Materials and Methods

### Strains and media

R265, a VGIIa clinical *C*. *gattii* isolate from the Vancouver Island outbreak[4], was used as a background strain in this study. Mutant and complemented strains of R265 were constructed by biolistic transformation. All of the strains were maintained on non-selective yeast extract peptone dextrose solid medium (YPD, 1% yeast extract, 2% peptone, 2% dextrose, and 2% agar). Selective media containing geneticin (G418) or nourseothricin were used for the screening of mutant or reconstituted strains as previously reported[23].

### Construction of mutant and reconstituted *C*. *gattii* strains

The primers used in this study are listed in S1 Table. For gene deletion, overlap PCR was employed to generate the knock-out cassette of *Cg-UBP5*, including the flanking fragments and *NEO* resistance gene[53]. The purified PCR products were precipitated onto gold microparticles and introduced into R265 cells by biolistic transformation[13]. Stable transformants were screened using selective medium that included G418 and then confirmed via diagnostic PCR, DNA sequencing, and Southern blot analysis. Southern blot examination was performed as previously described[23].

A whole DNA fragment of *Cg-UBP5* containing the ORF, promoter and terminator region was amplified from R265 genomic DNA for mutant complementation. The purified PCR fragments were linked to the digested plasmid pCH233 by Xba I using the Infusion®EcoDry^TM^ Cloning System (Clontec, Mountain View, US). The linked fragments were then introduced into the mutants by biolistic transformation. Stable colonies were screened on selective medium containing nourseothricin, and finally confirmed by diagnostic PCR and Southern blot analysis.

### *In vitro* phenotypic assays

The yeast cells were cultured to saturation in YPD broth at 30°C, washed twice with 1×PBS buffer, and then quantified using a Countstar Automated Cell Counter. To evaluate the stress response of *C*. *gattii* strains, the cells were serially diluted 10-fold (1–10^6^ dilutions). 3 μL suspension of 10^8^ cells/mL were spotted on different stress media, incubated for five days and then photographed. For the high temperature stress test, the yeast cells were incubated at 30°C, 37°C and 39°C on YPD agar. For the oxidative and NO stress test, the cells were incubated on Yeast Nitrogen Base (YNB) agar containing 2 mM H_2_O_2_ or 0.75 mM NaNO_2_ (pH 4.0). To evaluate the response to high salt and osmotic stress, 1.5 M NaCl, 1.5 M KCl or 1.5 M sorbitol was added to the YPD agar. To assess cell wall/membrane integrity stress, 0.5% Congo Red or 0.02% SDS was added to the YPD agar.

The antifungal susceptibility test was performed as previously reported[23]. The MICs of common antifungal agents (including amphotericin B, flucytosine, terbinafine, and azoles) against the R265, *Cg-ubp5*Δ, and *Cg-ubp5*Δ*+UBP5* strains were determined using the Clinical and Laboratory Standards Institute broth microdilution reference method (CLSI, 2002), and *Candida parapsilosis* ATCC22019 served as a quality control strain.

To measure capsule production, fungal cells were incubated on DMEM medium for three days in the presence of 5% CO_2_ at 37°C[54]. The capsule was stained with India ink and visualized by microscopy. The relative capsule volume was calculated for at least 50 cells for each strain according to the following formula: (Total Volume–Packed Volume)/Total Volume. To analyze melanin production, a 5 μL suspension of 10^7^ cells/mL for each strain was spotted on L-DOPA and Caffeic Acid medium[55] and then incubated for 5 days at 30°C.

### Macrophage killing assays

J744.A1. macrophage cells were used to assay the intracellular survival of different *C*. *gattii* strains as previously described[23, 56]. In brief, each strain was incubated overnight at 30°C and then opsonized with cryptococcal monoclonal antibody (C66441M, bought from Meridian Life Science, Inc. Saco, US). A total of 10^6^ yeast cells were co-incubated with 10^5^ J744.A1. cells that had been activated with interferon-gamma and lipopolysaccharide in 96-well tissue culture plates. After a 2-hour co-culture, the extracellular yeasts were washed away with PBS buffer, and fresh DMEM medium was added. After 24 hours of incubation, the macrophages were lysed with 0.5% SDS, and viable fungal cells were calculated by quantitative culture on YPD agar at 30°C for 3 days.

### Virulence assays *in vivo*

BALB/c mice were intranasally infected according to an established protocol[23]. For the survival assay, ten mice per group were challenged with 10^5^ CFU of the mutant (*Cg-ubp5*Δ), wild-type(WT) (R265), or complemented (*Cg-ubp5*Δ+*UBP5*) strain in 50 μL of PBS. All of the mice were monitored daily for signs of infection and sacrificed via carbon dioxide euthanasis based on predetermined endpoints such as weight loss (≥15%), neurological symptoms, and an inability to access food or water. To assess the organ fungal burden, lungs and brains were removed from the sacrificed mice (12 mice per group) after 3, 7, 14, and 21 days.

A *Caenorhabditis elegans (C*. *elegans)* model was also used to evaluate the virulence of each strain under room temperature as previous reported[27]. Briefly, a total of 40 standard *C*. *elegans* strain N2 Bristol in each group were incubated to the young adult developmental stage on a lawn of *Escherichia coli* OP50. Subsequently, they were transferred to plates containing WT, mutant, or complemented strains. The viability of *C*. *elegans* was determined every day by microscopy.

### Statistics

The data obtained for the mouse and *C*. *elegans* model survival assays were plotted as Kaplan-Meier survival curves and analyzed with the log-rank test using SPSS 18.0 software. The LF50 (time for half of the worms to die) was also calculated to estimate survival differences in the *C*. *elegans* model. The remaining statistical analyses were conducted with the student’s *t* test or Mann-Whitney test. The results were considered statistically significant when the *P* value was less than 0.05.

### Ethics Statement

The animal studies were proved by the Committee on Ethics of Biomedicine Research, Second Military Medical University, and carried out in strict accordance with the recommendations in the Regulations for the Administration of Affairs concerning Experimental Animals of the State Science and Technology Commission (China). Animal model was established under isofluorane anesthesia, and all efforts were made to minimize animal suffering and distress.

## Supporting Information

S1 Fig Phylogenetic tree analysis of Ubp5 orthologs. The alignment of predicted Ubp5 orthologs from various fungal species was performed using the DNASTAR 6.13 ClustalW multiple-sequence alignment. The organism sources and accession numbers (NCBI database) for the protein sequences are as follows: *C*. *gattii* R265, KGB80315; *C*. *gattii* WM276, XP_003197136; *C*. *neoformans* var. *grubii* (*CnVG*) H99, AFR99081; *C*. *neoformans* var. *neoformans* (*CnVN*) JEC21, XP_572460; *Ustilago maydis*, XP_758786; *Candida albicans*, KGQ89526; *Clavispora lusitaniae*, XP_002617519; *Candida glabrata*, XP_449943; *Saccharomyces cerevisiae*, EWH16885; *Aspergillus fumigatus*, XP_748018; *Talaromyces marneffei*, XP_002147746; *Colletotrichum fioriniae*, XP_007599895; *Fusarium graminearum*, XP_009255591; *Mucor circinelloides*, EPB84371.
(TIF)

S1 Table Primers used in this study. (DOC)
