# Supplementary figures and images for: Deubiquitinase Ubp5 Is Required for the Growth and Pathogenicity of Cryptococcus gattii
Source: PLoS One. 2016 Apr 6;11(4):e0153219. doi: 10.1371/journal.pone.0153219 (PMC4822882; doi:10.1371/journal.pone.0153219)

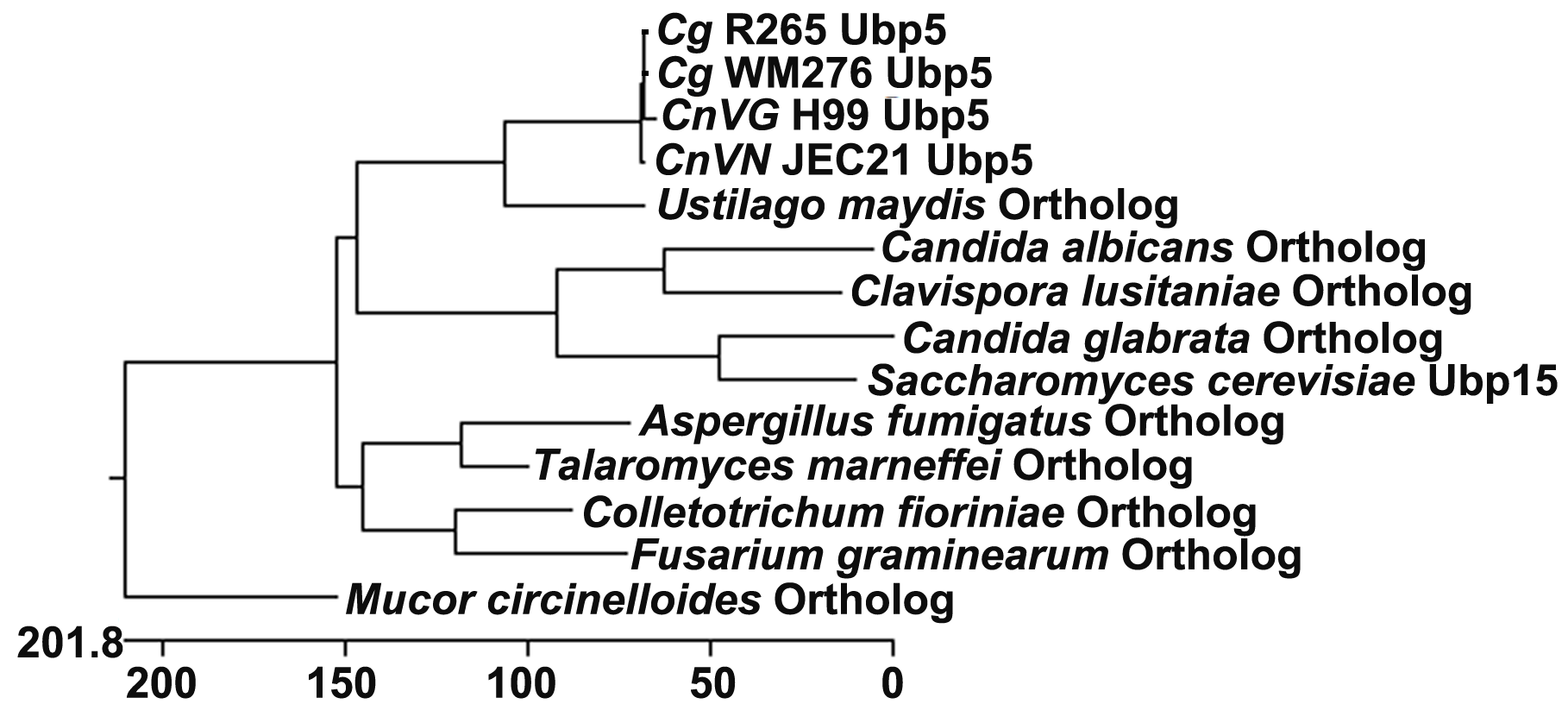

Supplement: S1 Fig — The alignment of predicted Ubp5 orthologs from various fungal species was performed using the DNASTAR 6.13 ClustalW multiple-sequence alignment. The organism sources and accession numbers (NCBI database) for the protein sequences are as follows: C. gattii R265, KGB80315; C. gattii WM276, XP_003197136; C. neoformans var. grubii (CnVG) H99, AFR99081; C. neoformans var. neoformans (CnVN) JEC21, XP_572460; Ustilago maydis, XP_758786; Candida albicans, KGQ89526; Clavispora lusitaniae, XP_002617519; Candida glabrata, XP_449943; Saccharomyces cerevisiae, EWH16885; Aspergillus fumigatus, XP_748018; Talaromyces marneffei, XP_002147746; Colletotrichum fioriniae, XP_007599895; Fusarium graminearum, XP_009255591; Mucor circinelloides, EPB84371. (TIF) [file pone.0153219.s001.tif]
