# Supplementary material for: Deubiquitinase Ubp5 Is Required for the Growth and Pathogenicity of Cryptococcus gattii
Source: PLoS One. 2016 Apr 6;11(4):e0153219. doi: 10.1371/journal.pone.0153219 (PMC4822882; doi:10.1371/journal.pone.0153219)
Supplement: S1 Table — (DOC) [file pone.0153219.s002.doc]

**S1 Table. Primers used in this study.**

| **Primer** | **Sequence (5’->3’)** | **Purpose** |
| --- | --- | --- |
| CgUBP5-5UTR-F | TTTTTCAATGTCAGCAACAG | Construction of knockout fragment |
| CgUBP5-5UTR-R | GTCATAGCTGTTTCCTGGATATGAAGGGCAAATGTGT | Construction of knockout fragment |
| CgUBP5-3UTR-F | CTGGCCGTCGTTTTACTATTTTTGGATGCTCGAAGT | Construction of knockout fragment |
| CgUBP5-3UTR-R | GAATTGTTTCGTCCATCAGT | Construction of knockout fragment |
| M13F | GTAAAACGACGGCCAG | Construction of knockout fragment |
| M13R | CAGGAAACAGCTATGAC | Construction of knockout fragment |
| CgUBP5-Probe-F | ACTAATGTGCCTCGTTCTTT | Screening the reconstituted strain *Cg-ubp5∆+UBP5* |
| CgUBP5-Probe-R | TCCCCAATCACATTCTTCAG | Screening the reconstituted strain *Cg-ubp5∆+UBP5* |
| CgUBP5-DP-F | CTCGCCATCAAATGCTAA | Screening the *Cg-ubp5∆* mutant |
| CgUBP5-DP-R | GATTCTGGGAGGTAAACG | Screening the *Cg-ubp5∆* mutant |
